# Paranasal Sinus Neuroendocrine Carcinoma: A Case Report and Review of the Literature

**DOI:** 10.1155/2013/728479

**Published:** 2013-02-14

**Authors:** Nagesh T. Sirsath, K. Govind Babu, Umesh Das, C. S. Premlatha

**Affiliations:** ^1^Department of Medical Oncology, Kidwai Memorial Institute of Oncology, Bangalore 560029, Karnataka, India; ^2^Department of Pathology, Kidwai Memorial Institute of Oncology, Bangalore 560029, Karnataka, India

## Abstract

Neuroendocrine neoplasms are defined as epithelial neoplasms with predominant neuroendocrine differentiation. They can arise in almost every organ of the body although they are most commonly found in the gastrointestinal tract and respiratory system. Nasal cavity and paranasal sinuses are a rare site for neuroendocrine carcinoma. In contrast to the other regions, neuroendocrine tumours of the sinuses have been reported to be recurrent and locally destructive. Very few cases of paranasal sinus neuroendocrine carcinoma have been reported till date. Difficulty in pathologic diagnosis and rarity of this malignancy have hindered the progress in understanding the clinical course and improving outcomes. We herein report a case of poorly differentiated neuroendocrine tumour of ethmoid and sphenoid sinus with invasion of orbit and intracranial extension. The patient had complete response at the end of chemoradiation and he was disease-free for 9 months duration after which he developed bone metastasis without regional recurrence.

## 1. Introduction

 In the nasal and paranasal sinus regions, squamous cell carcinoma is the most common tumor, followed by adenocarcinoma, malignant lymphoma, sinonasal undifferentiated carcinoma, malignant melanoma, and olfactory neuroblastoma [1]. Primary sinonasal neuroendocrine carcinomas are rare and represent a histological spectrum of differentiation. Neuroendocrine neoplasms are classified into well-differentiated (typical carcinoid), moderately differentiated (atypical carcinoids), and poorly differentiated (small and nonsmall cell types). Well-differentiated and, to a lesser extent, moderately differentiated neuroendocrine carcinomas carry better prognosis. Small-cell neuroendocrine carcinoma (SNEC), that is, poorly differentiated neuroendocrine carcinoma, was first described in the 19th century in the context of lung cancer. Head and neck SNEC have been described only since 1965 [2]. Poorly differentiated sinonasal neuroendocrine carcinoma is an extremely rare and aggressive neoplasm with a high recurrence rate and a tendency to metastasize to other sites via the lymphatic system and blood stream [3]. Because of the rarity of sinonasal neuroendocrine carcinoma, no agreement for adequate management has been reached among oncologists. The purpose of this paper is to analyse available information regarding this uncommon malignancy. The epidemiology, clinical features, pathological findings, differential diagnosis, and evolution of treatment of this rare neoplasm will be discussed. The paper presents recent treatment trends that may result in improved locoregional control and survival.

## 2. Case Report

**Figure 1:**
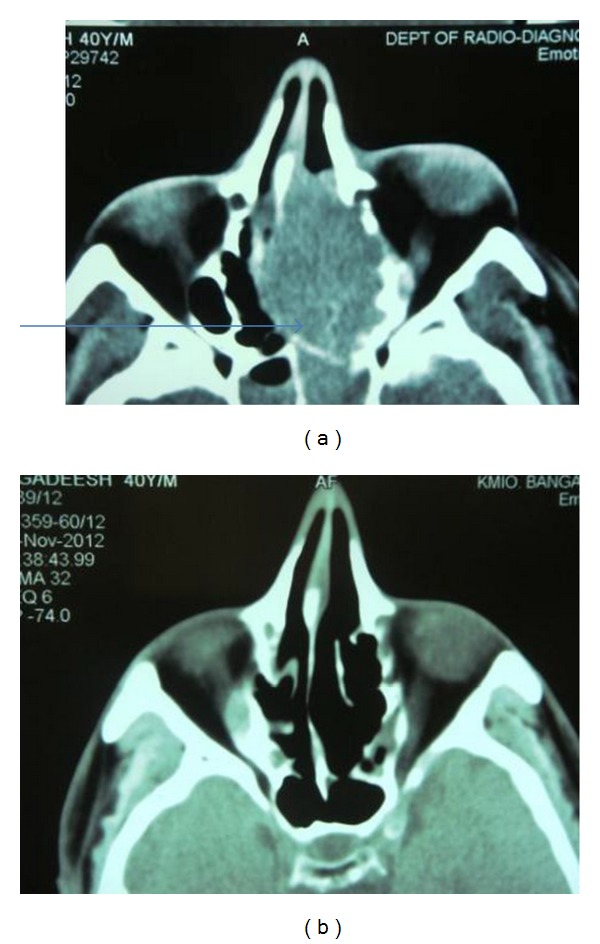
Computed tomography (CT) of paranasal sinuses and orbit showing mass (arrow) occupying the entire left ethmoid and sphenoid sinus extending into left nasal cavity causing erosion of nasal septum, extending into orbit and intracranial extension into basifrontal area (before chemoradiation and after chemoradiation).

**Figure 2:**
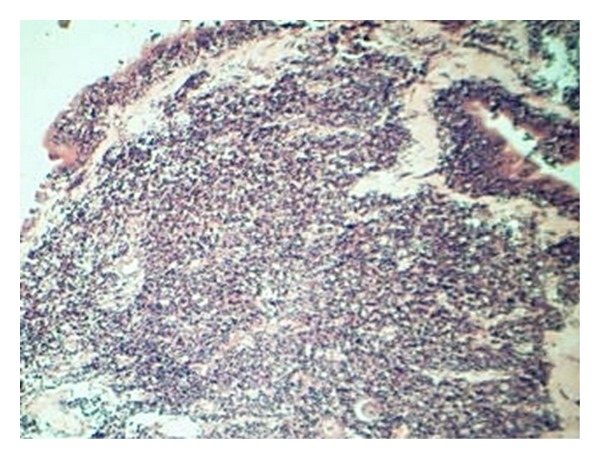
(haematoxylin and eosin ×100): Showing poorly differentiated malignant neoplasm with neuroendocrine differentiation.

**Figure 3:**
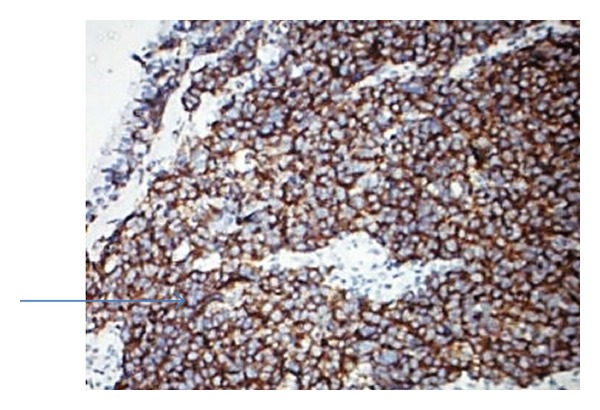
Neuroendocrine cells showing synaptophysin positivity (arrow). (Immunoperoxidase technique, HPR polymerase method ×100.)

A 40-year male presented with bleeding from left nasal cavity and mild proptosis of left eye for 1-month duration. On examination, he had gross deviation of nasal septum with a pinkish mass in left nasal cavity. Computed tomography (CT) of paranasal sinuses and orbit revealed soft-tissue mass occupying the entire left ethmoid and sphenoid sinus extending into left nasal cavity causing erosion of nasal septum, extending into orbit and intracranial extension into basifrontal area (Figure 1(a)). Biopsy from nasal mass was reported as poorly differentiated malignant neoplasm with neuroendocrine differentiation (Figure 2). Immunohistochemistry (IHC) revealed that neoplastic cells were positive for cytokeratin, synaptophysin, and chromogranin and negative for S-100 (Figure 3). Mitotic count was 30–40/10 hpf. A diagnosis of poorly differentiated neuroendocrine carcinoma of ethmoid and sphenoid sinus with invasion of orbit and intracranial extension was made. In view of intracranial extension, surgery as a primary treatment could not be offered and the patient received 2 cycles of induction chemotherapy (cisplatin and etoposide). There was gross reduction in tumour volume but intracranial extension was still persistent. Patient further received 3DCRT (conformal radiotherapy) followed by two more cycles of cisplatin and etoposide. Reassessment showed that the tumour had regressed completely (Figure 1(b)). Patient was on regular follow up and disease-free for 9 months duration after which he started back pain. Bone scan revealed skeletal deposits in calvaria, S1 vertebra, and sacroiliac joint. Computed tomography (CT) of head, neck, thorax, and abdomen revealed that there was no evidence of local recurrence and any other distant failure. The patient is receiving palliative radiotherapy for bone metastasis.

## 3. Discussion

 Sinonasal neuroendocrine carcinoma was first proposed as an entity by Silva et al. in 1982 [5]. During the past 40 years, 75 cases of small cell neuroendocrine carcinoma of the nasal and paranasal cavities have been reported in the literature. The mean age of 20 patients reviewed by Silva et al. [5] was 50 years with equal number of males and females. The authors failed to find any correlation between the occupations of the patients and the occurrence of neuroendocrine carcinoma. Of the 6 cases of sinonasal small cell neuroendocrine carcinoma reported by Perez-Ordonez et al. [6], there were 3 females and 3 males with a mean age at presentation of 51 years (range, 38 to 68). They failed to find any co-relation between EBV infection and occurrence of sinonasal neuroendocrine carcinoma. Babin et al. [7] reported 21 cases of sinonasal neuroendocrine tumour, 12 were male, and 9 were female, with a mean age at presentation of 55 years (range, 27 to 79). Likhacheva et al. [8] reviewed 20 patients treated for neuroendocrine carcinoma of the nasal cavity or paranasal sinuses from 1992 to 2008 at MD Anderson Cancer Centre; 11 were male and 9 female with a median age of 49.2 years. Han et al. [9] in their review of the previous 54 cases of small cell neuroendocrine carcinoma of the nasal and paranasal cavity reported a male predominance, with a male/female ratio 1.6 : 1, and a mean age of 51.3 years. Mitchell et al. [4] in their review of 28 patients with paranasal sinus neuroendocrine carcinoma had 16 males and 12 females with a median age of 56 years. Unlike other types of carcinoma, such as squamous cell carcinoma, which are seen most commonly in maxillary sinuses, paranasal sinus neuroendocrine carcinoma is most common in ethmoid sinuses [4, 9]. A strong linkage with smoking has not been identified in neuroendocrine carcinoma of the paranasal sinuses [4, 10]. From these observations it is evident that neuroendocrine carcinoma of sinonasal tract occurs slightly more commonly in males and is more prevalent in 5th and 6th decades. Till now no specific etiologic factor has been identified.

 The clinical features of sinonasal neuroendocrine carcinoma are nonspecific and similar to those of other sinonasal tumors. Common presentations include nasal obstruction, epistaxis, facial mass, and/or facial pain. Majority of patients have advanced disease at presentation [4]. Extensive involvement including the skull, orbit, and brain may be seen. Ophthalmic manifestations include exophthalmos, visual acuity trouble, and limitation in eye mobility. Local pain, anosmia, and metastatic cervical nodes have also been described [5]. The most frequent sites for distant metastases are the lungs, liver, and bone [5]. Analysis of the published literature reveals that these tumours are recurrent and locally destructive. Association between small cell neuroendocrine carcinoma and adenocarcinoma of the nasal cavity has been reported [5]. Babin et al. [7] reported one case of SNEC associated with an inverted papilloma. Vasan et al. [11] and Rossi et al. [12] have reported neuroendocrine carcinoma of the nasal cavity disclosing a syndrome of inappropriate antidiuretic hormone secretion (SIADH). After successful chemotherapy and radiotherapy treatment for the neoplasm, SIADH resolved. Kameya et al. [13] in their morphological and endocrinological study of neuroendocrine carcinoma of the paranasal sinus found elevated plasma levels of cortisol and adrenocorticotropic hormone associated with adrenocortical hyperplasia in one patient while another case showed hypercalcemia with bone metastasis, hypercalcitoninemia with a high content of calcitonin in the tumor tissue. 

 Most authors have relied on Kadish et al. staging system [14] and 2002-American Joint Committee on Cancer Staging system of nasal cavity and paranasal sinus tumors. (Kadish A: limited to nasal cavity, Kadish B: limited to nasal cavity and paranasal sinuses, Kadish C: tumour extending beyond the nasal cavity and paranasal sinuses).

 The tumours present with a variety of different histological patterns, including organoid, trabecular, cords, sheets, ribbons, pseudoglands and rosette formations, cribriform, solid, and single-file patterns. Lymph-vascular, perineural, and soft tissue invasion is common. The degree of cellular pleomorphism, mitotic activity, and necrosis increases as the tumour becomes more poorly differentiated (small cell carcinoma). Grimelius' argentic staining spots cytoplasmic neurosecretory granulation, which reveals the neuroendocrine characteristic of the carcinoma. This coloration is positive in 80% of the cases. Immunocytochemistry involves a carcinoma marker containing cytokeratine, and neuroendocrine differentiation is based on markers containing chromogranin, synaptophysin, and neuron-specific enolase. 

Sinonasal neuroendocrine carcinoma has to be differentiated from other neoplasms involving nasal cavity and paranasal sinuses such as squamous cell carcinoma, lymphoma, melanoma, olfactory neuroblastoma, and sinonasal undifferentiated carcinoma. Conventional microscopy is generally insufficient for arriving at accurate diagnosis and immunohistochemistry studies are invariably needed. Sinonasal squamous cell carcinoma has a male predilection (2 : 1), with a peak incidence in the sixth-seventh decades; it involves mostly maxillary sinus. It can be either keratinizing or nonkeratinizing. In keratinizing type, tumour cells exhibit squamous differentiation, keratinization, and variable degree of nuclear anaplasia. The nonkeratinizing type of SCC forms solid nests of variable sizes, frequently with relatively smooth borders. Sinonasal lymphomas can be excluded by lack of expression of leucocyte common antigen (LCA). Sinonasal melanomas usually express S-100 and HBM-45. Recently, Melan-A has been widely used in the diagnosis of melanomas.  Table 1 illustrates histological and immunohistochemical features of sinonasal tumours with neuroendocrine differentiation. In the present case, synaptophysin and chromogranin were positive while S-100 was negative which favoured the diagnosis of neuroendocrine carcinoma over olfactory neuroblastoma. 

The treatment of sinonasal neuroendocrine carcinomas has not been systematically evaluated because of small number of cases. No agreement for adequate management has been reached among oncologists, and some recommendations have been developed from retrospective data. Surgery, radiotherapy, and chemotherapy alone or in combination have been used in the past for the patients with NEC of the paranasal sinuses and nasal cavity.

**Figure 4:**
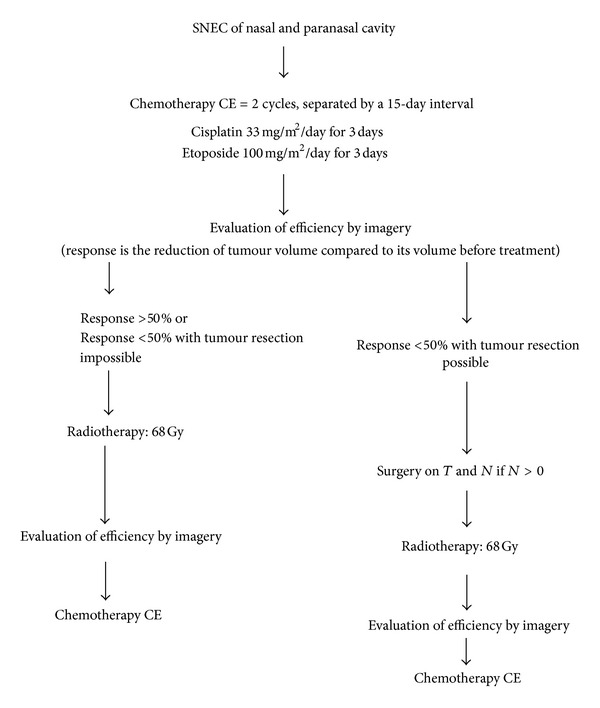
Treatment protocol of nasal and paranasal cavities small cell neuroendocrine carcinoma.

In the 1980s, surgery followed by radiotherapy was the routine approach to treat small cell tumours. Perez-Ordonez et al. [6] have emphasized the use of combined-modality therapy for these neoplasms. In the late 1990s, Fitzek et al. [15] and Bhattacharyya et al. [16] showed promising results with induction chemotherapy with cisplatin and etoposide followed by radiation in treatment of these tumors. Dramatic response was obtained even in bulky or unresectable disease. Babin et al. [7] formulated treatment protocol for sinonasal neuroendocrine tumour based on promising results given by studies conducted by Bhattacharyya et al. [16], Fitzek et al. [15] and after the results of the 35th Congress of the French Cervico-Faciale Carcinologic Society, Poitiers, France (November 2003) (Figure 4).

 Although Bhattacharyya et al. [16], Fitzek et al. [15], and Babin et al. [7] have proposed chemotherapy followed by radiation with surgery reserved for nonresponders as treatment protocol for sinonasal neuroendocrine carcinomas, a few recent studies have shown that surgery as an initial treatment followed by postoperative chemoradiotherapy is associated with better disease control and overall survival in treatment of sinonasal neuroendocrine carcinoma even in poorly differentiated small cell neuroendocrine carcinoma. Chang et al. [17], Qian et al. [18], and Likhacheva et al. [8] have concluded that combined treatment based on surgery is associated with significantly better disease-free survival and overall survival as compared to treatment without surgery irrespective of differentiation status of tumour. Because of intracranial extension, we could not offer the benefit of surgery to our patient and he was treated with chemoradiation.

## 4. Conclusion

Paranasal sinus neuroendocrine tumours are recurrent and locally destructive. Multimodality treatment approach is needed. Even with multimodality treatment, local and distant recurrence is very high. Platinum-based chemotherapy followed by radiotherapy has shown promising results in treatment of poorly differentiated small cell neuroendocrine carcinomas involving sinonasal tract. Recent treatment modality incorporating surgery as initial treatment followed by postoperative chemoradiotherapy is associated with better disease control and overall survival in treatment of sinonasal neuroendocrine carcinoma even in poorly differentiated small cell neuroendocrine carcinoma; however as majority of patients have advanced disease at presentation with extensive involvement of orbit, skull, and brain, surgical resection is usually difficult.

## Figures and Tables

**Table 1:** Histological and immunohistochemical features of sinonasal tumours with neuroendocrine differentiation [4].

Tumor classification	Histology				IHC				
	Morphology	Nucleoli	Mitotic	Other	K	SP	CG	S-100	NF
ENB	Small cells sheets	Absent	Low	Homer-Wright rosette, fibrillary cytoplasm	−	+	+	+	+
SNUC	Large cells sheets, nests	prominent	High	Necrosis/no squamous or glandular differentiation	+	−	−	−	−/+
NEC	Small cells sheets, ribbons	Absent	High	Necrosis	+	+	−/+	−	−

ENB: esthesioneuroblastoma, SNUC: sinonasal undifferentiated carcinoma, NEC: neuroendocrine carcinoma, K: keratin, SP: synaptophysin, CG: chromogranin, NF: neurofilaments.
